# The association between insured male expatriates’ knowledge of health insurance benefits and lack of access to health care in Saudi Arabia

**DOI:** 10.1186/s12889-018-5293-0

**Published:** 2018-03-15

**Authors:** Abdulwahab A. Alkhamis

**Affiliations:** College of Health Sciences, Saudi Electronic University, PO Box 15271, Riyadh, 11444 Saudi Arabia

**Keywords:** Access to health care, Knowledge of health insurance, Saudi Arabia, Expatriates, Insurance

## Abstract

**Background:**

Insufficient knowledge of health insurance benefits could be associated with lack of access to health care, particularly for minority populations. This study aims to assess the association between expatriates’ knowledge of health insurance benefits and lack of access to health care.

**Methods:**

A cross-sectional study design was conducted from March 2015 to February 2016 among 3398 insured male expatriates in Riyadh, Saudi Arabia. The dependent variable was binary and expresses access or lack of access to health care. Independent variables included perceived and validated knowledge of health insurance benefits and other variables. Data were summarized by computing frequencies and percentage of all quantities of variables. To evaluate variations in knowledge, personal and job characteristics with lack of access to health care, the Chi square test was used. Odds ratio (OR) and 95% confidence interval (CI) were recorded for each independent variable. Multiple logistic regression and stepwise logistic regression were performed and adjusted ORs were extracted.

**Results:**

Descriptive analysis showed that 15% of participants lacked access to health care. The majority of these were unskilled laborers, usually with no education (17.5%), who had been working for less than 3 years (28.1%) in Saudi Arabia. A total of 23.3% worked for companies with less than 50 employees and 16.5% earned less than 4500 Saudi Riyals monthly ($1200). Many (20.3%) were young (< 30 years old) or older (17.9% ≥ 56 years old) and had no formal education (24.7%). Nearly half had fair or poor health status (49.5%), were uncomfortable conversing in Arabic (29.7%) or English (16.7%) and lacked previous knowledge of health insurance (18%). For perceived knowledge of health insurance, 55.2% scored 1 or 0 from total of 3. For validated knowledge, 16.9% scored 1 or 0 from total score of 4. Multiple logistic regression analysis showed that only perceived knowledge of health insurance had significant associations with lack of access to health care ((OR) = 0.393, (CI) = 0.335–0.461), but the result was insignificant for validated knowledge. Stepwise logistic regression gave similar findings.

**Conclusions:**

Our results confirmed that low perceived knowledge of health insurance in expatriates was associated with less access to health care.

## Background

The positive association between obtaining insurance and access to health care has been reported in literature [1, 2]. However, there are people who face challenges in understanding how health insurance works and how to estimate out-of-pocket costs. These people, even if insured, are at risk of being without needed care [3] or experiencing delayed access to health care [4]. Poor understanding of health insurance is associated with delayed [4] or reduced care [5, 6], care avoidance [3, 7], poor health status [8] and low efficiency of the health care sector [9]. However, most of these studies were conducted in older populations such as Medicare recipients in the United States or did not include minorities in their studies.

There is evidence from different contexts that the minority populations are more likely to have a poor understanding of health insurance schemes [10–13]. However, these studies did not investigate the degree to which expatriates’ or minorities’ lack of health insurance knowledge influenced their access to health care.

In Saudi Arabia, expatriate workers represent more than 80% of private sector employees and comprise 56% of the total workforce [14, 15]. The dominance of expatriate workers mainly in the private sector incentivized the government to mandate that the employers in the private sector shoulder the health care expenses of their expatriate employees while ensuring access to health care [16]. Saudi Arabia introduced the Cooperative Health Insurance Law in 1999. The law not only mandates that all employers bear the full amount of their employees’ health insurance premium but it also determines the unified benefits packages, which are managed by a government body called the Council of the Cooperative Health Insurance (CCHI) [17]. The CCHI regulates and monitors the cooperative health insurance implementation so that all minimum medical treatments and other medical needs are determined and unified under one insurance policy [17]. Before the implementation of cooperative health insurance,^1^ there was no clear method for expatriates to access health care services or determine how much and who was responsible for paying for their health care expenses [16]. Health care expenses and coverage vary among employees; some pay the full amount out of pocket but others pay nothing [16].

In 2016, more than 12 million people (38% of the total population of Saudi Arabia) were insured through the cooperative health insurance [18]. Among these, more than 9.4 million (78%) were expatriates. The gross written insurance premiums were more than 36 billion Saudi Riyals ($9.6 billion). Of this total more than 16 billion Saudi Riyals ($4.26 billion) were for health insurance [19]. Although insurance companies are one of the main sources responsible for increasing public knowledge of health insurance [20], the insurance sector in Saudi Arabia is still in the development stage [21]. Furthermore, insurance companies have been the most reported for non-compliance by customers as reported by CCHI, since the first CCHI report in 2007 [18, 22–30]. When insured people are not knowledgeable about health insurance, this raises questions about the seriousness of policy makers and the effectiveness of health insurance companies in communicating with their customers [7].

In addition, the majority of expatriate workers (85%) are in laborer positions and low-skilled jobs [15]. They are less-educated people and are likely to have low knowledge about health insurance [31]. These factors raise the question of the influence of knowledge of health insurance benefits scheme against expatriates’ access to health care. There was not any previous study that investigates the association of knowledge of health insurance benefits with lack of access to health care in Saudi Arabia. This study will focus on the influence of perceived and validated knowledge on lack of access to health care of insured expatriates in Saudi Arabia.

## Methods

Face-to-face surveys were administered by trained bilingual research assistants to 3398 male expatriate workers in the private sector, with a 96% response rate. The survey was conducted orally to minimize language and literacy barriers. One of the main criteria for selecting the research assistants was their ability to speak the expatriates’ main languages (Arabic, Urdu, Bengali, Hindi, Malayalam, Nepali, and Tagalog), in addition to their communication skills, mobility among different companies and comprehension of the questionnaire.

The questionnaire was initially developed in English. The final version was translated into the expatriates’ main languages and verified using backward translation methods.

Cross-sectional survey data were collected by trained bilingual research assistants in Riyadh. Riyadh was selected as the setting for the study because the Riyadh region contains around 30% of Saudi Arabia’s total expatriate population and more than one-fourth of the Saudi population [32, 33].

The data were collected between March 2015 and February 2016 at the participants’ work sites with permission from their employers. Using a multi-stage stratified cluster-sampling method, the data were collected based on business sector, company size, and number of employees as determined by the Ministry of Labor and Social Development [34]. The sampling approach used in this study was adapted from another study conducted in the same setting [34]. Expatriates working in the health care and health insurance sectors were excluded from the study because their health insurance knowledge and health care access would likely be affected by vocational factors.

The questionnaire consisted of sections on health insurance status, access to health care, knowledge of health insurance (including perceived knowledge and validated knowledge), employment information, the employee’s previous knowledge of insurance (in their home country) and demographic data.

### Statistical analysis

In this study, the dependent variable is binary, expressed as “0,” indicating access, and “1” indicating no access. This coding is intended to facilitate analysis so as to predict lack of access to health care. The question assessed the lack of access to health care during the preceding 1 year. The question was adapted from the 2015 Medical Expenditure Panel Survey (MEPS) [35] and also has been adapted by two other studies measuring access to health care for expatriates and immigrants [16, 36].

The main independent variables were perceived and validated overall knowledge scores. These knowledge questions were scored as follows: incorrect responses were given a zero score, while 1 point was given to each correct answer; a correct response being that based on current literature. The knowledge scores were then computed by totaling the number of correct answers.

The following questions were used to obtain information on perceived knowledge: “Does your health insurance cover outpatient care (such as doctor visits)?”, “Does your health insurance cover inpatient care (including surgery)?” and “Does your health insurance cover prescription drugs?” The maximum perceived knowledge score was 3.

Four questions were used to assess verified knowledge: “Do you know how much your copayment is for seeing a general practitioner as an outpatient?”, “Do you know how much the maximum payment is when visiting an outpatient doctor?”, “Do you know how much you have to pay for inpatient services if you are admitted to hospital?” and “Do you know how much you have to pay for prescription drugs?” The maximum score for verified knowledge was 4. Other independent variables were also considered, including personal and job characteristic variables. The full descriptions of each category have been elaborated in a previous study by Alkhamis [10].

Data were summarized by computing frequencies and percentages of all qualitative variables. To evaluate knowledge and demographic and job characteristic variables associated with lack of access to health care, the Chi square test was used. In addition, odds ratio (OR) and 95% confidence interval (CI) were recorded for each independent variable. Multiple logistic regressions in which all independent variables are forced into the model was estimated and adjusted ORs were extracted from this model. Stepwise multiple logistic regressions were also performed to determine the most important predictors of lack of access to health care. All of the tests for significance were two-sided and *p* values < 0.05 were considered statistically significant. All analyses were done using Statistical Package for the Social Sciences (SPSS) version 22 for Windows (SPSS Inc., Chicago, USA) software.

## Results

The total number of participants was 3398 but 280 surveyed participants were excluded because they were not in Saudi Arabia during their last sickness which reduced the total number of participants who answered questions on access to medical care to 3118. Table 1 includes the characteristics of study participants (personal and job features), the percentage lacking access to health care, their previous knowledge of health insurance and their existing knowledge of health insurance benefits (perceived and validated knowledge).

**Table 1:** The main descriptive analysis and number of expatriates with lack of access to health care based on their personal characteristics, jobs characteristics, previous knowledge and existing knowledge (including perceived and validated knowledge)

	Number	Number of participants lack access to health care (%)
Job characteristics of participants		
Position in your company		
Specialist with university education	664	86(13)
Professional with education level higher than high school	367	39(10.6)
Technical with high school education	365	53(14.5)
Manual laborer with less than high school education	883	149(16.9)
Unskilled usually with no education	836	146(17.5)
Total	3115	473(15.2)
Length of working in Saudi Arabia		
Less than one year	225	78(34.7)
One year but less than three years	431	106(24.6)
Three years but less than five years	623	99(15.9)
Five to ten years	1393	117(8.4)
More than ten years	446	73(16.4)
Total	3118	473(15.2)
How many employees are there in your company?		
< 50	1422	331(23.3)
> =50	1696	142(8.4)
Total	3118	473(15.2)
What kind of business/industry does your company do?		
Industrial	322	56(17.4)
Construction	1164	94(8.1)
Trade	781	130(16.6)
Other	851	193(22.7)
Total	3118	473(15.2)
What is your usual monthly income including all allowances?		
Less than 2000 S.R.	1418	251(17.7)
Between 2001 – 4500 S.R.	1113	167(15)
Between 4501 – 6000 S.R.	448	45(10)
Between 7501 – 9000 S.R.	138	10(7.2)
Total	3117	473(15.2)
Personal characteristics of participants		
Age Group		
18 to 30	867	176(20.3)
31 to 45	1898	253(13.3)
46 to 55	282	28(9.9)
56 years old and above	56	10(17.9)
Total	3103	467(15)
Nationality		
Asian	2483	330(13.3)
Arabs	625	141(22.6)
Others	7	1(14.3)
Total	3115	472(15.2)
Highest Educational Attainment		
Illiterate	85	21(24.7)
Elementary	1403	228(16.3)
High School	486	81(16.7)
High Education (Diploma & above)	1143	143(12.5)
Total	3117	
Marital Status		
Single	467	65(13.9)
Married	2593	395(15.2)
Divorced/Wido*w*/widower	21	5(23.8)
Total	3081	473(15.2)
What is your native language?		
Arabic	620	136(21.9)
Urdu	832	127(15.3)
Hindi	684	90(13.2)
Malayalam	118	16(13.6)
Bengali	287	39(13.6)
Tagalog	259	18(6.9)
Others	311	46(14.8)
Total	3111	472(15.2)
Are you comfortable conversing in English?		
No	1443	241(16.7)
Yes	1670	231(13.8)
Total	3113	472(15.2)
Are you comfortable conversing in Arabic?		
No	576	171(29.7)
Yes	2533	299(11.8)
Total	3109	470(15.1)
Health Status		
Excellent and very good	2689	336(12.5)
Good	332	92(27.7)
Fair and poor	91	45(49.5)
Total	3112	473(15.2)
Previous Knowledge		
No	1156	208(18.0)
Yes	1962	265(13.5)
Total	3118	473(15.2)
Score of perceived knowledge		
0	241	144(59.8)
1	214	107(50)
2	754	100(13.3)
3	1909	122(6.4)
Total	3118	473(15.2)
Score of validated Knowledge		
0	974	289(29.7)
1	1588	145(9.1)
2	180	16(8.9)
3	163	15(9.2)
4	213	8(3.8)
	3118	473(15.2)

The descriptive analysis in Table 1 shows that the insured participants with lack of access to health care represent 15% of the total sample. The same table also shows that majority of participants (63%) knew about insurance before coming to Saudi Arabia. Participants who scored 3 out of 3 in perceived knowledge were 61%, whereas only 6.8% scored 4 out of 4 for validated knowledge about health insurance.

Table 1 provides the profile of expatriates who encountered problem in accessing health care.

The expatriates were either young (< 30 years old; 20.3%) or old (≥ 56 years old; 17.9%) and had no formal education (24.7%). They reported fair or poor health status (49.5%), were uncomfortable conversing in Arabic (29.7%) or English (16.7%) and had no previous knowledge of health insurance before coming to Saudi Arabia (18.%). More than half of them (55.2%) scored 1 or 0 out 3 for perceived knowledge and 1 or 0 out of 4 for validated knowledge (16.9%). Table 1 also indicates that majority of the expatriates were unskilled laborers, usually with no education (17.5%) and had been working for less than 3 years (28.1%) in Saudi Arabia. A total of 23.3% worked for companies with less than 50 employees and 16.5% earned less than 4500 Saudi Riyals monthly ($1200). In companies with less than 50 employees, 23.3% of expatriates do not have access to health care which implies that 76.7% of expatriates working in companies with less than 50 employees are able to access health care.

Table 2 shows the crude OR using simple logistic regression and adjusted logistic regression using multiple logistic regressions by forcing all variables (main personal, job characteristics and perceived and existing knowledge of health insurance benefits variables) into the equation. This indicated a significant association between personal and job characteristics, as well perceived and validated knowledge about health insurance benefit and lack of access to health care. However, after adjusting for the lack of access to health care, logistic regression analysis shows lack of access to health care only has significant associations with perceived knowledge of health insurance (OR = 0.393, CI = 0.335–0.461), companies with more than 50 employees (OR = 0.455, CI = 0.343–0.603), comfort in conversing in Arabic (OR = 0.471, CI = 0.340–0.652) and excellent health status (OR = 0.241, CI = 0.141–0.411), or good health status (OR = 0.438, CI = 0.245–0.783).

**Table 2:** Summary of simple and multiple logistical regression analysis of the influence of knowledge of health insurance, workplace and personal characteristics on lack of access to health care

	CRUDE ODDS RATIO		ADJUSTED ODDS RATIO	
The main parameters	Odds ratio	95% of confidence interval	Odds Ratio	95% of confidence interval
Position in your company				
Unskilled usually with no education	Reference		Reference	
Specialist with university education	0.7032*	(.527–.938)	1.343	(.830–2.174)
Professional with education level higher than high school	0.5619*	(.385–.819)	0.822	(.515–1.312)
Technical with high school education	0.8028	(.571–1.130)	1.02	(.638–1.631)
Manual laborer with less than high school education	0.9594	(.747–1.233)	0.988	(.723–1.348)
Length of working in Saudi Arabia				
Less than one year	Reference		Reference	
One year but less than three years	0.6147*	(.433–.873)	1.163	(.740–1.827)
Three years but less than five years	0.3561*	(.251–.505)	1.151	(.728–1.821)
Five to ten years	0.1728*	(.124–.241)	0.911	(.570–1.454)
More than ten years	0.3688*	(.254–.535)	1.018	(.605–1.713)
How many employees are there in your company?				
< 50	Reference		Reference	
> = 50	0.3012*	(.244–.372)	0.455*	(.343–.603)
What kind of business/industry does your company do?				
Other	Reference		Reference	
Industrial	0.7178*	(.516–.998)	1.209	(.791–1.849)
Construction	0.2995*	(.230–.390)	0.915	(.639–1.308)
Trade	0.6808*	(.532–.872)	0.87	(.616–1.228)
What is your usual monthly income including all allowances?				
Less than 2000 S.R.	Reference		Reference	
Between 2001 – 4500 S.R.	0.8208	(.821–1.016)	0.903	(.656–1.243)
Between 4501 – 6000 S.R.	0.5192*	(.371–.727)	0.724	(.446–1.175)
Between 7501 – 9000 S.R.	0.3632*	(.188–.701)	0.818	(.359–1.863)
Age Group				
56 years old and above	Reference		Reference	
18 to 30	1.1716	(.580–2.368)	0.533	(.234–1.215)
31 to 45	0.7075	(.353–1.420)	0.463	(.210–1.024)
46 to 55	0.5071	(.231–1.114)	0.516	(.213–1.251)
Nationality				
Asian	Reference		Reference	
Arabs	1.901*	(1.525–2.369)	1.993	(.460–8.643)
Others	1.087	(.130–9.061)	1.117	(.071–17.533)
Highest Educational Attainment				
Illiterate	Reference		Reference	
Elementary	0.5914*	(.354–.988)	1.056	(.552–2.021)
High School	0.6095	(.353–1.054)	0.914	(.447–1.869)
High Education (Diploma & above)	0.4358*	(.258–.735)	1.043	(.498–2.186)
Marital Status				
Single				
Married	1.111	(.837–1.475)	1.247	(.883–1.761)
Divorced/widow/widower	1.933	(.685–5.456)	1.390	(.298–6.476)
What is your native language?				
Arabic	Reference		Reference	
Urdu	0.6411*	(.490–.838)	3.628	(.848–15.513)
Hindi	0.5392*	(.403–.722)	3.083	(.713–13.335)
Malayalam	0.5582*	(.319–.978)	2.558	(.556–11.767)
Bengali	0.5597*	(.380–.825)	4.166	(.932–18.627)
Tagalog	0.2658*	(.159–.445)	2.324	(.484–11.155)
Others	0.6178*	(.428–.891)	2.29	(.516–10.152)
Are you comfortable conversing in English?				
No	Reference		Reference	
Yes	0.8006*	(.658–.974)	0.862	(.644–1.154)
Are you comfortable conversing in Arabic?				
No	Reference		Reference	
Yes	0.3170*	(.255–.393)	0.471*	(.340–.652)
Health Status				
Fair and poor	Reference		Reference	
Excellent and very good	0.1460*	(.095–.224)	0.241*	(.141–.411)
Good	0.3919*	(.243–.631)	0.438*	(.245–.783)
Previous Knowledge				
No	Reference		Reference	
Yes	0.712*	(.584–0.868)	0.903	(.686–1.189)
Perceived Knowledge	0.33*	(.298–.366)	0.393*	(.335–.461)
Validated Knowledge	0.44*	(.379–.511)	0.911	(.774–1.073)

*p-value is significant at < 0.05

Table 3 shows the results of stepwise logistic regression to determine the most important predictors of lack of access to health care. These findings are similar to the adjusted OR in Table 2 including the ability to converse in English language as an associated factor influencing lack of access to health care (OR = 0.755, CI = 0.597–0.954). Moreover, the OR and CI values are similar to the adjusted ORs and CIs in Table 2. Table 3 illustrates that variables perceived knowledge, health status, company size, comfort conversing in English and comfort conversing in Arabic are the most important predictors of lack of access to health care for insured expatriates.

**Table 3:** Summary of stepwise logistic regression of the influence of knowledge of health Insurance, personal and workplaces characteristics on lack of access to health care

The main parameters	Odds ratio	95% of confidence interval
Perceived knowledge	0.395*	(.353–.442)
Health Status		
Fair and poor	Reference	
Excellent/Very good	0.219*	(.132–.363)
Good	0.394*	(.225–.693)
Company size		
< 50	Reference	
> = 50	0.493*	(.385–.632)
Are you comfortable conversing in English?		
No	Reference	
Yes	0.755*	(.597–.954)
Are you comfortable conversing in Arabic?		
No	Reference	
Yes	0.396*	(.304–.515)

*p-value is significant at < 0.05

## Discussion

This is the first study investigating the association between expatriate workers’ knowledge of health insurance benefits and lack of access to health care in Saudi Arabia. This study further assesses expatriates’ previous knowledge of insurance before their arrival to Saudi Arabia as an influencing factor on health insurance knowledge.

To measure knowledge of health insurance benefits, expatriate workers were asked what they thought they knew about their health insurance coverage (perceived knowledge) and compared their answers to the actual health insurance coverage they have (validated knowledge) based on the official existing health insurance benefits scheme implemented for expatriate workers in Saudi Arabia. The study showed a significant association between perceived knowledge and lack of access to health care in both adjusted and unadjusted ORs. Low scores for perceived knowledge of health insurance benefits indicate a likelihood of expatriates not accessing health care. This finding might explain the findings of a study by Alkhamis [16] showing that uninsured but paying expatriates (either cash or via reimbursement) had better access to health care than some insured expatriates. Those studies did not consider the knowledge of health insurance benefits as study variables.

The current study stresses that it is not necessary to include numerical knowledge of health insurance benefits such as the amount of the copayments as part of knowledge of main health insurance benefits. Expatriates would want to know their health insurance coverage rather than details of their copayments. This interesting finding could be related to the nature of the CEBHI, in which beneficiaries need not worry about the exact amount of maximum copayment since it is unified regardless of health insurance class (e.g. VIP, class A, Class B).

Remarkably, this study did not find most personal/socioeconomic characteristics, such as income, education, and age, to be detrimental factors to lack of access to health care. This is consistent with another study which reported the availability of health insurance is more important for access to health care than other immigrant socioeconomic factors such as income or education level [36]. The Jine Choi study showed that, compared with Filipino and Korean immigrants, although Marshallese immigrants had lower socioeconomic status including lower income and health education, they had better access to health care because they had the highest health insurance enrollment rate among the three groups. However, as reported by Alkhamis [10], socioeconomic factors like income and education are important contributing factors in increasing expatriates’ knowledge of health insurance benefits.

The effects of these variables, for example, are indirectly associated with access to health care. Income and education levels influence expatriates’ knowledge of health insurance benefits although the level of direct influence on access to health care was found not significant. Although this study identified four variables associated with lack of access to health care, it is well documented that other variables contribute indirectly to expatriates’ lack of access to health care such as personal and job characteristics, have been associated with expatriates’ knowledge of health insurance [10].

The current study finds inability to comfortably speak Arabic has significant association with lack of access to health care, which is consistent with a previous study [36]. This finding cannot be isolated from the influence of length of stay in Saudi Arabia. Expatriates who work in Saudi Arabia for longer periods have greater opportunities to improve their language ability and increase their ability to converse in Arabic.

The interrelationships between these variables are complex and difficult to segregate their influence from one another. Whereas some variables have direct influence on lack of access to health care, other variables have indicated an indirect influence on lack of access to health care.

Finally, the role of employers in increasing their expatriate employees’ access to health care is very important, as supported by studies from different contexts [37], whether by increasing their knowledge of health insurance benefits [10] or by increasing their access to health insurance [34].

This study was cross-sectional in design, which limited our ability to investigate causal inferences. Although we have included many variables in our analysis, it is possible that a variable associated with both correlated variables was omitted or an omitted variable correlated with both. However, our multivariate analyses included a large number of relevant covariates with a large sample size.

In addition, the cross-sectional study did not improve our understanding of the chronological order of events. In other words, it is hard to know whether expatriates’ knowledge was associated with their lack of access to health care or if the lack of access to health care had affected their knowledge of health care. However, given the correlation between these parameters identified in this study, future studies could investigate the details of this relationship.

Another limitation is self-reported data on lack of access to health care, which is subject to recall bias. Recall bias could be a major issue in particular if the research participants are old. However, the expatriate population of Saudi Arabia is young; 61% of our sample was under 46 years old. In addition, recall by younger respondents for general questions about health care access has been reported to be accepted [38, 39]. Furthermore, the measures of health care access used in this study have been used with similar populations before [16, 36]. For the above mentioned reasons the recall biased has been mitigated. The study used only one question to measure lack of access to health care. However, other studies have used the same measure of access for similar populations and did not report problems related to the use of one question to assess access to health care [16, 36]. Another study has characterized MEPS as one of the most reliable questionnaires for measuring access to health care [40]. Another limitation of the study is that only adult males were surveyed. Women and children were omitted from this study because female expatriates represent only 2% of the private sector [41] and the majority of female expatriates work in health care [33], which is one of the sectors excluded from the study.

## Conclusion

The results of this study show that there is a significant association between insured expatriates’ knowledge of health insurance benefits and their lack of access to health care. More specifically, this study indicates that low perceived knowledge of health insurance among expatriates was associated with less access to health care. Interestingly, expatriates’ validated knowledge about health insurance benefits was not significantly associated with lack of access to health care after adjustment for other variables. This study stresses the importance of expatriates’ knowledge of health insurance benefits as opposed to knowledge of numerical copayment details.

In addition, the study illustrates that insurance availability for expatriates is not enough to guarantee their access to health care because lack of knowledge of health insurance benefits is significantly associated with lack of access to health care. The study findings cannot be isolated from the complex and interconnected relationships between different variables.

## Abbreviations

CCHI: Council of the cooperative health insurance
CI: Confidence interval
MEPS: Medical expenditure panel survey
OR: Odds ratio
SPSS: Statistical package for the social sciences

## Footnotes

1 The official name is Cooperative Health Insurance although it is a mandatory health insurance scheme. This name was given ease its enactment and acceptance [10]. Technically, this scheme should have been called Compulsory Employment-Based Health Insurance as stated by other study [21].
